# Imaging of Endogenous Metabolites of Plant Leaves by Mass Spectrometry Based on Laser Activated Electron Tunneling

**DOI:** 10.1038/srep24164

**Published:** 2016-04-07

**Authors:** Lulu Huang, Xuemei Tang, Wenyang Zhang, Ruowei Jiang, Disong Chen, Juan Zhang, Hongying Zhong

**Affiliations:** 1 Mass Spectrometry Center for Structural Identification of Biological Molecules and Precision Medicine, Key Laboratory of Pesticides and Chemical Biology, Ministry of Education, College of Chemistry, Central China Normal University, Wuhan, Hubei 430079, P. R. China

## Abstract

A new mass spectrometric imaging approach based on laser activated electron tunneling (LAET) was described and applied to analysis of endogenous metabolites of plant leaves. LAET is an electron-directed soft ionization technique. Compressed thin films of semiconductor nanoparticles of bismuth cobalt zinc oxide were placed on the sample plate for proof-of-principle demonstration because they can not only absorb ultraviolet laser but also have high electron mobility. Upon laser irradiation, electrons are excited from valence bands to conduction bands. With appropriate kinetic energies, photoexcited electrons can tunnel away from the barrier and eventually be captured by charge deficient atoms present in neutral molecules. Resultant unpaired electron subsequently initiates specific chemical bond cleavage and generates ions that can be detected in negative ion mode of the mass spectrometer. LAET avoids the co-crystallization process of routinely used organic matrix materials with analyzes in MALDI (matrix assisted-laser desorption ionization) analysis. Thus uneven distribution of crystals with different sizes and shapes as well as background peaks in the low mass range resulting from matrix molecules is eliminated. Advantages of LAET imaging technique include not only improved spatial resolution but also photoelectron capture dissociation which produces predictable fragment ions.

**C**urrently, secondary ion mass spectrometry (SIMS)^1^,^2^, matrix-assisted laser desorption ionization (MALDI)^3^,^4^,^5^ and desorption electrospray ionization (DESI)^6^,^7^,^8^ imaging of biological samples have allowed scientists to obtain spatial distributions of a wide range of biological molecules in tissues that are critical for understanding of their physiological roles^9^,^10^,^11^. Among these three approaches, SIMS provides the best lateral resolution (~50 nm) so far. It has a long history for biological imaging but suffers from extensive fragmentations due to energetic desorption/ionization and limitations in the efficiency for secondary ion production. In contrast, MALDI is a soft ionization approach where pulsed laser beams are fired at matrix crystals with embedded analyzes. It offers spatial resolution in the level of micrometers which enables applications on the cellular or sub-cellular levels. However, background peaks in low mass region of time of flight (TOF) instrument, as well as serious contamination of ion source chamber resulting from organic matrix molecules limit its application for analysis of small molecules and other biological molecules. Nanostructure-initiator mass spectrometry (NIMS)^12^,^13^,^14^ has recently emerged as an alternative new method for MS imaging that does not need chemical matrix materials. The spatial resolution can be down to 150 nm. In this approach, initiator molecules are trapped in nanostructured surfaces or clathrates to release and ionize intact molecules adsorbed on the surface. Both NIMS and MALDI MS analyze biological samples in the vacuum. DESI is an atmospheric pressure technique that evaporates and ionizes molecules with a fine spray of charged droplets in the open air. It is well established for ambient surface analysis of every kind of samples such as metabolites, proteins, animal tissue sections, luggage screening for explosives as well as latent fingerprints. The major challenge of DESI is the spatial resolution for biological applications. Additionally, in the case of analysis of plants, the surface wax layer and strong cell wall prevent the penetration of the spray of liquid droplets.

We herein describe the new application of a newly developed soft ionization approach based on laser activated electron tunneling (LAET)^15^,^16^,^17^ from semiconductor nanoparticles of bismuth cobalt zinc oxide. These nanoparticles not only can absorb UV irradiation but also have high electron mobility. Although nanoparticles such as gold, titanium dioxide, carbon nanotubes and zinc oxide have been used as inorganic matrix materials for MALDI and SALDI MS (surface assisted laser desorption ionization mass spectrometry) analysis for many years^18^,^19^,^20^,^21^,^22^, research efforts have been focused only on their roles as energy mediators. It has been widely accepted that nanoparticles have high surface areas, high photo absorption capabilities and can effectively transfer photo energy to surrounding analyze molecules, which are eventually ionized and detected in positive ion mode of mass spectrometers^23^,^24^,^25^. This work is aimed to demonstrate the roles of photo-generated electrons in ionization of biological molecules. It is applied as a new avenue for imaging of endogenous metabolites present in plant leaves.

## Methods

### Reagents and apparatus

LC-MS grade water, acetonitrile (ACN), ethanol and isopropanol were purchased from Fisher Scientific (Bridgewater, NJ, USA). Nanoparticles of bismuth cobalt zinc oxide (Bi_2_O_3_)_0.07_(CoO)_0.03_(ZnO)_0.9_ (<100 nm BET or <50 nm XRD), gibberellic acid (GA), salicylic acid (SA), jasmonic acid (JA), indoleacetic acid (IAA) and abscisic acid (ABA) were purchased from Sigma-Aldrich (St. Louis, MO, USA). Free fatty acids including C6:0, C8:0, C10:0, C12:0, C14:0, C16:0, C18:0, C20:0 and C22:0 were purchased from NU-CHEK PREP, Inc (Elysian, MN, USA) for instrumental calibration. Aluminum tape was purchased from Junke (Shanghai, China).

### Preparation of compressed thin films of bismuth cobalt zinc oxide

Nanoparticles of bismuth cobalt zinc oxide (Bi_2_O_3_)_0.07_(CoO)_0.03_(ZnO)_0.9_ were thermally treated at 350 °C for 2 hours in a muffle furnace made by Jianli Furnace Co. Ltd (Yingshan, Hubei, China) before use in order to remove trace organic contaminants. Then about 10 mg of those treated nanoparticles were put on the sticky surface of a single-sided adhesive aluminum tape and compressed under 10 MPa of pressure for 2 minutes by a compressor that is regularly used in infrared spectroscopic analysis. By using this approach, a very uniform and tight flat thin film of bismuth cobalt zinc oxide can be obtained.

### Preparation of plant leaves

Freshly obtained plant leaves (*Cayratia japonica)* were tightly pressed onto the top of the thin film of bismuth cobalt zinc oxide. It is very critical for leaves to be tightly attached to the surface of bismuth cobalt zinc oxide. Otherwise, no signals can be obtained. In order to make sure that there is no air gap between the leaf and the flat thin film, the leaf was compressed under 0.1 MPa pressure against the film. Glue tapes were attached to short stems in order to fix the position of the leaves on the film. Then the aluminum tap was finally taped on the sample plate.

### Mass spectrometric analysis

A Waters Synapt G2 HDMS system (Billerica, MA, USA) with a MALDI source was used for mass spectrometric imaging. Data acquisition for visualization of spatial distribution of negatively charged molecular ions and fragment ions was obtained through moving the sample plate. The mass spectrometer is equipped with an Nd: YAG high repetition laser head (355 nm). Laser spot size is adjustable from 5 μm to 250 μm. In this work, laser spot size was fixed at ~15 μm. Laser influx (355 nm) has been set as 200 units. Laser pulse width is 3 ns and laser fire rate was set as 200 Hz. Pulse energy is 100 μJ/200 Hz. For each pixel, the acquisition time is 1 second. Potential difference between the sample plate and the aperture were set as 20 volts. Voltages on the sample plate and aperture are 87 volts and 107 volts respectively for this work. In negative ion mode, the instrument was internally calibrated with standard solution of free fatty acids.

For quantitative assessment of the proposed approach, nanoparticles of semiconductors were suspended in a pure isopropanol solution to reach a concentration of ~10 mg/ml and then pipetted into sample wells of the sample plate. Gibberellic acid, salicylic acid, jasmonic acid, indoleacetic acid and abscisic acid were dissolved in a solution containing 50% (v/v) acetonitrile and 50% (v/v) ethanol to reach a concentration of 100 mg/mL. After nanoparticles on the sample plate were air dried, 1 μL of standard solutions of fatty acids, or samples was deposited on the surface of nanoparticles for downstream mass spectrometric analysis.

For mass spectrometric imaging analysis, the Waters software MALDI Imaging Pattern Creator (Billerica, MA, USA) was used to define the imaging areas before subject to laser shot. The step size of laser beams scanning across the leaf was set as 250 μm × 250 μm. Decreased step size improves spatial resolution but it takes longer time to establish the imaging. In the case of leaf analysis, long analysis time may cause dehydration of leaves. Taken together throughput and resolution, the step size of 250 μm × 250 μm was used for all imaging experiments of leaves.

Putative identification of plant endogenous metabolites was achieved by accurate masses and MS/MS fragment ions. As for high abundant molecules, MS/MS experiments have been performed. But only MS experiments have been performed for low abundant molecules. Images of ions have been compared with that of spontaneously generated fragment ions in order to validate the identities of observed ions. Mass spectrometric data have been searched against METLIN database (http://metlin.scripps.edu).

## Results and Discussion

### Principles of LAET ionization, fragmentation and mass spectrometric imaging

As shown in Supplementary Figure 1, nanoparticles of bismuth cobalt zinc oxide were compressed into a uniform thin film under 10 MPa pressure. The thin film is then stuck on the surface of a conductive sample plate. Voltages applied on the sample plate, extraction plate, hexapole and aperture were indicated as V0, V1, V2 and V3 respectively. Upon the irradiation of 355 nm UV laser pulses (3.5 eV, 3 ns width), electrons are excited from valence bands (VB) to conduction bands (CB), leaving holes behind. In negative ion mode of MALDI mass spectrometer, nanoparticles and samples were lined up in the same direction as that of the electric field present in the ion source chamber. Therefore the recombination of electron-hole pairs can be efficiently inhibited because these electrons and holes move in inverse directions and are instantly separated by the external electric field. With appropriate energies, photoexcited electrons can resonantly tunnel away from the surface barrier and eventually captured by charge deficient atoms present in neutral molecules through a nonergodic process. Because the pressure in ion source chamber of the mass spectrometer is usually around 6 × 10^−4^ mbar, highly reactive oxygen radical ions usually observed in photocatalysis processes cannot be formed. Photoelectron capture dissociation is demonstrated by the negative ion of long chain fatty acid C16:0 at m/z 255 shown in Supplementary Figure 2A. This ion was generated through the capture of a tunneling electron by the charge deficient carbon atom of the carboxyl group. Resultant unpaired-electron subsequently directs the cleavage of α O-H bond (Supplementary Figure 2B). Localization of charge deficient atoms and prediction of electron-directed chemical bond cleavage can be achieved with DFT calculation.

Compared with general MALDI approach, there are no background peaks from bismuth cobalt zinc oxide in the low mass region by using LAET-based point electron emitting sources for *in situ* soft ionization. In addition, the thin film of bismuth cobalt zinc oxide particles provides a uniform distribution of matrix nanoparticles. These features make LAET an ideal imaging technique for displaying the distribution of different molecules. Supplementary Figure 3 represents the plot of absolute mass spectrometric intensities of molecular ion at m/z 209.1196 of jasmonic acid versus different quantities. It was found that absolute intensities of molecular ions are correlated well with quantities. These experimental results indicate that intensities of each pixel in a LAET mass spectrometric imaging should be able to reflect the quantities of analyzes in different locations. Then it is possible to compare ion distribution in different tissue sections. Supplementary Figure 4 shows that flavone derivatives are not present in the roots but fatty acid C16:0 is highly abundance in the same sections. However, it should be mentioned that ion suppression resulting from co-existing molecules still limits the application to absolute quantification. Other phytohormones and organic acids have also been studied. For all these compounds, tunneling electrons are captured by charge deficient carbon atoms of carbonyl groups and unpaired-electrons cause subsequent cleavage of α O-H bond. As an example, production of molecular ion at m/z 209 of representative jasmonic acid was shown in Supplementary Figure 5. In this approach, fragmentation of molecules is controllable because the energy of emitting electrons is adjustable by changing the voltage applied on the sample plate. At low energies such as 20 eV used for routine MALDI experiments, emitting electrons are not able to cause intra-molecular vibrational excitation because the de Broglie wavelength of emitting electrons does not match the typical bond length of organic molecules. Therefore, the possibility to generate non-specific fragmentation observed in SIMS can be decreased.

**Figure 1:**
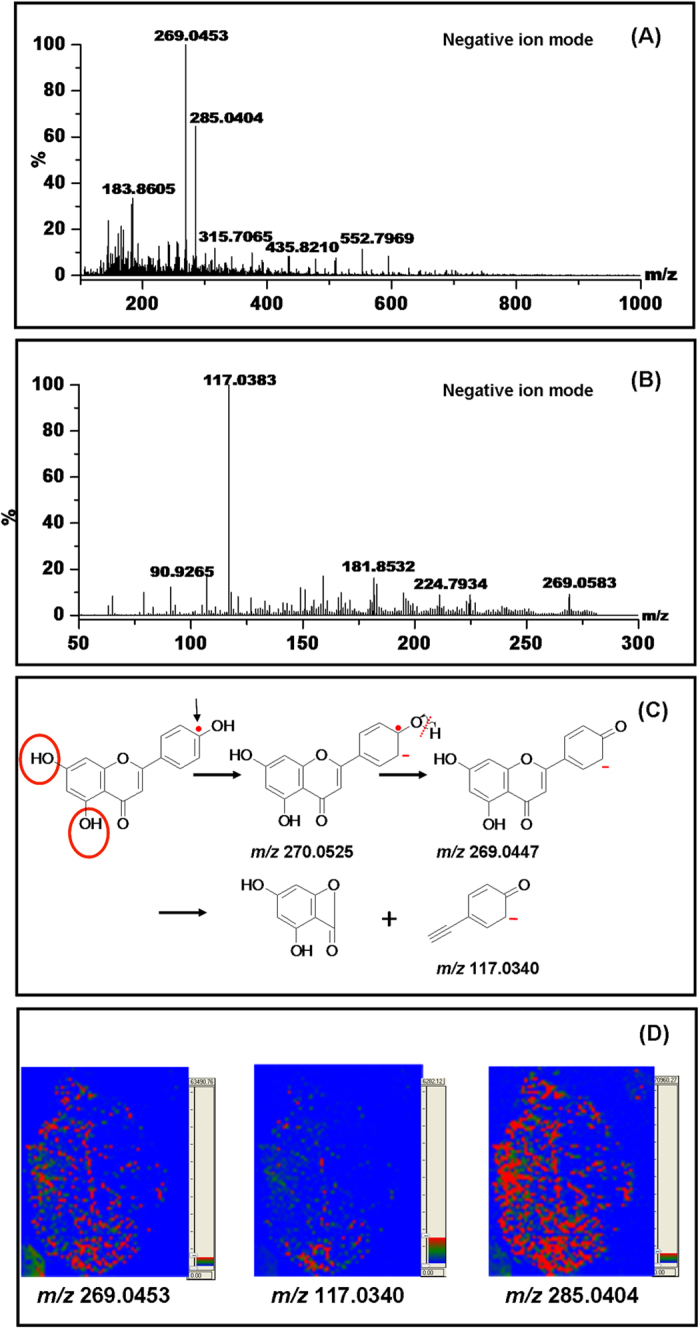
(**A**) Mass spectrum of the leaf stuck on the surface of bismuth cobalt zinc oxide film; (**B**) MS/MS spectrum of the selected ion at m/z 269.0453; (**C**) Illustration of the production of fragment ion at m/z 117.0340; (**D**) Mass spectrometric imaging of two putatively identified flavone derivatives at m/z 269.0453 and 285.0404 as well as the fragment ion at m/z 117.0340.

Actually LAET can work with two modes of fragmentation^16^,^17^. In addition to the unpaired electron-directed chemical bond cleavages, CAD (collision activated dissociation) can also be complemented for more detailed structural specificities. Figure 1(A) is the MS spectrum of the leaf. By searching molecular masses against METLIN database, two dominant peaks at m/z 269.0453 and 285.0404 have been putatively identified as flavone derivatives with three and four OH groups in benzoic rings respectively. Further MS/MS spectrum of the ion at m/z 269.0453 shown in Fig. 1(B) provides experimental evidences for the identification of this flavone derivative but the positions of two OH groups remain unknown. However, LAET can spontaneously generate fragment ions through electron-directed chemical bond cleavage without MS/MS experiments. The predictable fragmentation makes LAET an ideal tool for identification of small molecules. Figure 1(C) illustrates the production of the fragment ion at m/z 117.0340 and the possible positions of OH groups were indicated with red circles. Visualization of the distribution of these two flavone derivatives as well as the fragment ion at m/z 117.0340 was shown in Fig. 1(D). *Cayratia japonica* has been traditionally recognized as a Chinese medicinal herb. The presence of abundance flavone derivatives of this plant makes it useful to treat certain diseases^26^. Figure 1(D) indicates that this kind of natural products distributes across the whole leaf.

**Figure 2:**
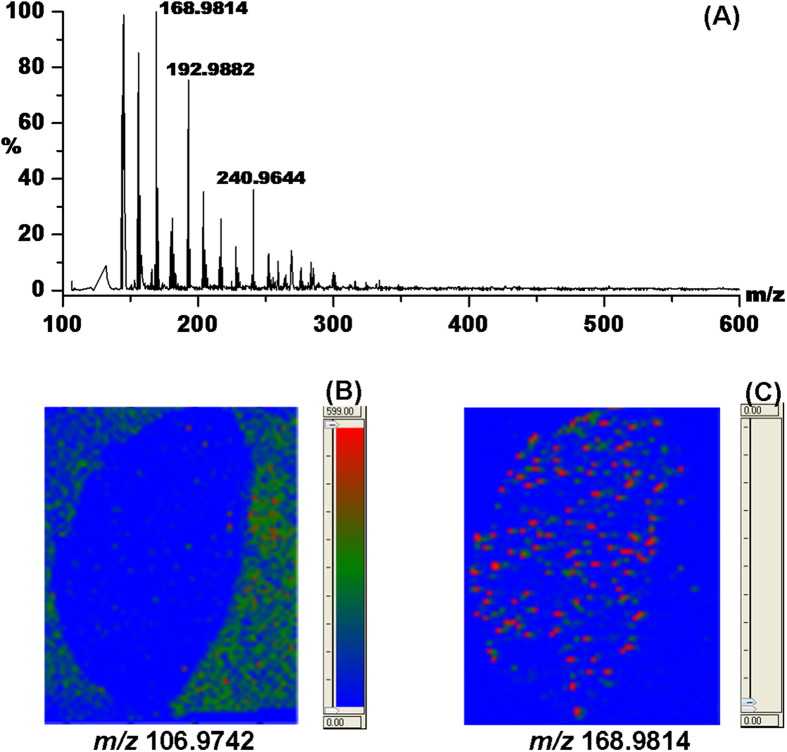
(**A**) Mass spectrum of the tape-blocked leaf stuck on the surface of bismuth cobalt zinc oxide film; (**B**) Mass spectrometric imaging of the ion at m/z 106.9742; (**C**) Mass spectrometric imaging of the ion at m/z 168.9814.

In order to proof that laser activated electron tunneling (LAET) from semiconductor nanoparticles initiate the ionization process, a tape has been stuck to the back of the leaf. Figure 2(A) shows the averaged MS spectrum of the tape-blocked leaf and several strong peaks have been observed. The imaging at m/z 106.9742 shown in Fig. 2(B) reveals that no signal can be detected except very weak background molecules around the leaf, meaning LAET cannot occurs on the surface of the tape. However, as shown in Fig. 2(C), even weaker molecules at m/z 168.9814 probably resulting from the ionization of the tape material can be observed in the area where the tape was stuck. These experimental results confirm that irradiation of ultraviolet laser on surfaces of semiconductor nanoparticles is essential to the generation of photoelectrons and subsequent electron-directed soft ionization of neutral molecules as well as chemical bond cleavages.

### Imaging of organic acids and phytohormones in plant leaves with LAET

**Figure 3:**
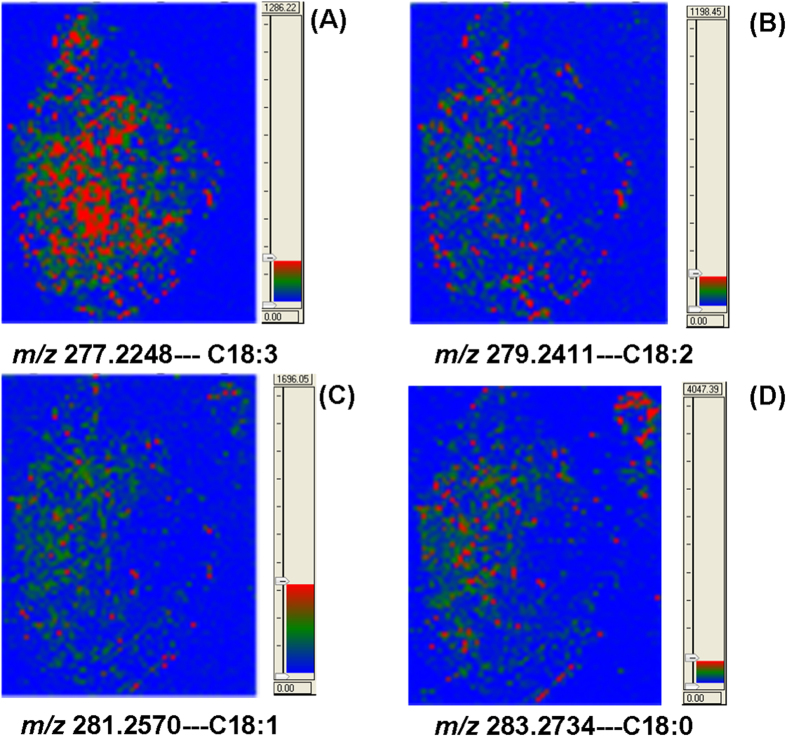
Mass spectrometric imaging of putatively identified long chain fatty acids including C18:3 (**A**), C18:2 (**B**), C18:1 (**C**) and C18:0 (**D**).

In addition to abundant flavone derivatives, other different ions have been detected with reasonable mass accuracy (Supplementary Table 1). Representative fatty acids, small organic acids and plant hormones were putatively identified by their masses (error < 35 ppm) and related fragment ions. No MS/MS experiments were performed for these endogenous metabolites because of their low abundance. The accuracy of detected molecular ions can be down to three decimals. Lower intensities of molecular ions usually result in decreased mass accuracy. Figure 3(A–D) shows the spatial distribution of fatty acids including C18:3, C18:2, C18:1 and C18:0. Other fatty acids have been shown in Supplementary Figure 6–21. The content of these fatty acids fluctuates across the whole section of the leaf. Among these, polyunsaturated long chain fatty acid C18:3 at m/z 277 shows relatively more uniform distribution while the others were found to be more abundant on the left side of the leaf. Uneven distribution of endogenous metabolites may result from nutritional differences, bias of sunlight, defenses or other physiological processes^27^,^28^,^29^,^30^.

**Figure 4:**
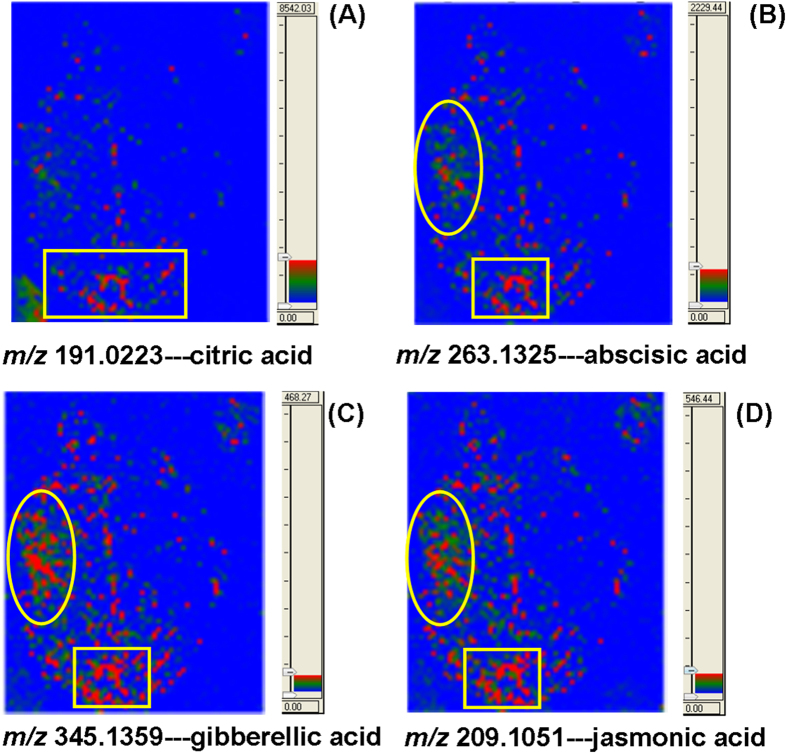
Mass spectrometric imaging of putatively identified small organic acids and phytohormones including citric acid (**A**), abscisic acid (**B**), gibberellic acid (**C**) and jasmonic acid (**D**).

In addition to long chain fatty acids, some small organic acids have also been detected. Figure 4(A) shows the spatial distribution of citric acid. It is interesting to find that this acid accumulates more around the stem where the leaf was cut. Small organic acids have been found in many plants and juice of fruits^31^,^32^,^33^,^34^. Tissue localization of these compounds is essential to investigate their versatile roles in every kind of physiological processes^35^. Phytohormones constitute another subclass of endogenous metabolites in plants that are important for immobile plants to sense and respond to environmental stresses^36^,^37^,^38^,^39^. Figure 4(B–D) shows different accumulation patterns for different phytohormones. The image of representative fragment ion of gibberellic acid (GA) was shown in Supplementary Figure 22. In general, these small signaling molecules are all unevenly distributed across the whole leaf. All of them were found to be more abundant in the left side of this leaf and around the stem where this leaf was cut. Among them, jasmonic acid (JA) and gibberellic acid (GA) were slightly more abundant in the middle of the left side. The interaction of salicylic acid (SA) and abscisic acid (ABA) has been reported in response to temperature changes. In plant cells, increased production of SA or ABA was considered as one of the consequences of temperature change senses and transduction of perceived signals^40^. Meanwhile, interactive effects of JA and GA on induction of trichomes in Arabidopsis have also been reported^41^,^42^.

## Conclusion

Compared with other approaches for direct tissue analysis, LAET offers the opportunities for endogenous metabolites of plants to be ionized and dissociated from biological context without background peaks. With LAET method, delocalization of analyzes caused by organic solvents in routine MALDI analysis is avoided because it is a solvent-free approach. The uniform surface of matrix nanoparticles simplifies the sample preparation process and enhances the spatial resolution as well as analytical reproducibility. It generates hot electrons *in situ* with adjustable energies for *in situ* soft ionization of biological molecules. Thus energetic fragmentations observed in high energy SIMS approach are avoided.

In summary, soft ionization of biological molecules on nanoparticles of bismuth cobalt zinc oxide is achievable. Atoms present in nanoparticles function as point electron emitting sources when laser activated electron tunneling occurs. Thermal electrons tunneling away from the surface can be captured by charge deficient atoms and unpaired electrons cause subsequent chemical bond cleavages. LAET provides a new and clean way for direct analysis of plant tissues.

## Additional Information

**How to cite this article**: Huang, L. *et al*. Imaging of Endogenous Metabolites of Plant Leaves by Mass Spectrometry Based on Laser Activated Electron Tunneling. *Sci. Rep*. **6**, 24164; doi: 10.1038/srep24164 (2016).

## Supplementary Material

Supplementary Information

## References

[b1] Slodzian G., Daigne B., Girard F., Boust F. & Hillion F. Scanning secondary ion analytical microscopy with parallel detection. Biol Cell 74, 43–50 (1992). 1511246 10.1016/0248-4900(92)90007-n

[b2] Delcorte A., Bour J., Aubriet F., Muller J. F. & Bertrand P. Sample metallization for performance improvement in desorption/ionization of kilodalton molecules: quantitative evaluation, imaging secondary ion MS, and laser ablation. Anal. Chem. 75, 6875–6885 (2003). 14670048 10.1021/ac0302105

[b3] Cornett D. S., Reyzer M. L., Chaurand P. & Caprioli R. M. MALDI imaging mass spectrometry: molecular snapshots of biochemical systems. Nat. Methods 4, 828–833 (2007). 17901873 10.1038/nmeth1094

[b4] Li Y., Shrestha B. & Vertes A. Atmospheric pressure infrared MALDI imaging mass spectrometry for plant metabolomics. Anal. Chem. 80, 407–420 (2008). 18088102 10.1021/ac701703f

[b5] Angel P. M., Spraggins J. M., Baldwin H. S. & Caprioli R. Enhanced sensitivity for high spatial resolution lipid analysis by negative ion mode matrix assisted laser desorption ionization imaging mass spectrometry. Anal. Chem. 84, 1557–1564 (2012). 22243218 10.1021/ac202383m PMC3277660

[b6] Takats Z., Wiseman J. M., Gologan B. & Cooks R. G. Mass spectrometry sampling under ambient conditions with desorption electrospray ionization. Science 306, 471–473 (2004). 15486296 10.1126/science.1104404

[b7] Ifa D. R., Manicke N. E., Dill A. L. & Cooks R. G. Latent fingerprint chemical imaging by mass spectrometry. Science 321, 805 (2008). 18687956 10.1126/science.1157199

[b8] Eberlin L. S., Ifa D. R., Wu C. & Cooks R. G. Three‐dimensional vizualization of mouse brain by lipid analysis using ambient ionization mass spectrometry. Angew. Chem. Int. Ed. 49, 873–876 (2010). 10.1002/anie.200906283 PMC2958060 20041465

[b9] Andersson M., Groseclose M. R., Deutch A. Y. & Caprioli R. M. Imaging mass spectrometry of proteins and peptides: 3D volume reconstruction. Nat. Methods 5, 101–108 (2008). 18165806 10.1038/nmeth1145

[b10] Huh W. K. . Global analysis of protein localization in budding yeast. Nature 425, 686–691 (2003). 14562095 10.1038/nature02026

[b11] Thomas A., Charbonneau J. L., Fournaise E. & Chaurand P. Sublimation of new matrix candidates for high spatial resolution imaging mass spectrometry of lipids: enhanced information in both positive and negative polarities after 1,5-diaminonapthalene deposition. Anal. Chem. 84, 2048–2054 (2012). 22243482 10.1021/ac2033547

[b12] Northen T. R. . Clathrate nanostructures for mass spectrometry. Nature 449, 1033–1036 (2007). 17960240 10.1038/nature06195

[b13] Woo H. K., Northen T., Yanes O. & Siuzdak G. Nanostructure-initiator mass spectrometry: a protocol for preparing and applying NIMS surfaces for high-sensitivity mass analysis. Nat. Protoc. 3, 1341–1349 (2008). 18714302 10.1038/nprot.2008.110

[b14] Greving M. P., Patti G. J. & Siuzdak G. Nanostructure-initiator mass spectrometry metabolite analysis and imaging. Anal. Chem. 83, 2–7 (2011). 21049956 10.1021/ac101565f PMC3012143

[b15] Zhong H., Fu J., Wang X. & Zheng S. Measurement of laser activated electron tunneling from semiconductor zinc oxide to adsorbed organic molecules by a matrix assisted laser desorption ionization mass spectrometer. Anal. Chim. Acta 729, 45–53 (2012). 22595432 10.1016/j.aca.2012.03.057

[b16] Tang X., Huang L., Zhang W. & Zhong H. Chemical imaging of latent fingerprints by mass spectrometry based on laser activated electron tunneling. Anal. Chem. 87, 2693–2701 (2015). 25647159 10.1021/ac504693v

[b17] Tang X., Huang L., Zhang W., Jiang R. & Zhong H. Photo-catalytic activities of plant hormones on semiconductor nanoparticles by laser-activated electron tunneling and emitting. Sci. Rep. 5, 8893 (2015). 25749635 10.1038/srep08893 PMC4352873

[b18] Watanabe T., Kawasaki H., YOnezawa T. & Arakawa R. Surface-assisted laser desorption/ionization mass spectrometry (SALDI-MS) of low molecular weight organic compounds and synthetic polymers using zinc oxide (ZnO) nanoparticles. J. Mass Spectrom. 43, 1063–1071 (2008). 18286665 10.1002/jms.1385

[b19] Lin Y. S., Tsai P. J., Weng M. F. & Chen Y. C. Affinity capture using vancomycin-bound magnetic nanoparticles for the MALDI-MS analysis of bacteria. Anal. Chem. 77, 1753–1760 (2005). 15762582 10.1021/ac048990k

[b20] Qiao L. . Specific on-plate enrichment of phosphorylated peptides for direct MALDI-TOF MS analysis. J. Proteome Res. 6, 4763–4769 (2007). 18047269 10.1021/pr0705284

[b21] Lorkiewicz P. & Yappert M. C. Titania microparticles and nanoparticles as matrixes for *in vitro* and *in situ* analysis of small molecules by MALDI-MS. Anal. Chem. 81, 6596–6603 (2009). 20337373 10.1021/ac9001113

[b22] Chen S. . Carbon nanodots as a matrix for the analysis of low-molecular-weight molecules in both positive- and negative-ion matrix-assisted laser desorption/ionization time-of-flight mass spectrometry and quantification of glucose and uric acid in real samples. Anal. Chem. 85, 6646–6652 (2013). 23796018 10.1021/ac401601r

[b23] Jackson S. N. . Imaging of lipids in rat heart by MALDI-MS with silver nanoparticles. Anal. Bioanal. Chem. 406, 1377–1386 (2014). 24309627 10.1007/s00216-013-7525-6 PMC5523126

[b24] Stubiger G., Wuczkowski M., Bicker W. & Belgacem O. Nanoparticle-based detection of oxidized phospholipids by MALDI mass spectrometry: Nano-MALDI approach. Anal. Chem. 86, 6401–6409 (2014). 24914456 10.1021/ac500719u

[b25] Kailasa S. K., D’souza S. & Wu H. F. Analytical application of nanoparticles in MALDI-MS for bioanalysis. Bioanal. 7, 2265–2276 (2015). 10.4155/bio.15.149 26354596

[b26] Han X. H. . Monoamine oxidase inhibitory components from *Cayratia japonica*. *Arch*. Pharm. Res. 30, 13–17 (2007). 10.1007/BF02977772 17328236

[b27] Shroff R., Vergara F., Muck A., Svatos A. & Gershenzon J. Nonuniform distribution of glucosinolates in *Arabidopsis thaliana* leaves has important consequences for plant defense. Proc. Natl. Acad. Sci. USA 105, 6196–6201 (2008). 18408160 10.1073/pnas.0711730105 PMC2329684

[b28] Holscher D. . Matrix-free UV-laser desorption/ionization (LDI) mass spectrometric imaging at the single-cell level: distribution of secondary metabolites of *Arabidopsis thaliana* and *Hypericum* species. Plant J. 60, 907–918 (2009). 19732382 10.1111/j.1365-313X.2009.04012.x

[b29] Hrazdina G., Marx G. A. & Hoch H. C. Distribution of secondary plant metabolites and their biosynthetic enzymes in pea (*Pisum sativum* L.) leaves : anthocyanins and flavonol glycosides. Plant Physiol. 70, 745–748 (1982). 16662568 10.1104/pp.70.3.745 PMC1065763

[b30] Gutterman Y. & Chauser-Volfson E. Secondary phenol metabolites (SPhMs), distribution and content of some Aloe species, originated from arid zones of South Africa: A review. Am. J. Food Technol. 2, 555–569 (2007).

[b31] Lynch J. M. Effects of organic acids on the germination of seeds and growth of seedlings. Plant cell Environ. 3, 255–259 (2006).

[b32] Melino V. J., Soole K. L. & Ford C. M. Ascorbate metabolism and the developmental demand for tartaric and oxalic acids in ripening grape berries. BMC Plant Biol. 9, 145 (2009). 19995454 10.1186/1471-2229-9-145 PMC2797797

[b33] Mukherjee M. . Ascorbic acid deficiency in Arabidopsis induces constitutive priming that is dependent on hydrogen peroxide, salicylic acid, and the NPR1 gene. Mol. Plant Microbe Interact. 23, 340–351 (2010). 10.1094/MPMI-23-3-0340 20121455

[b34] Sun Y. L. & Hong S. K. Effects of citric acid as an important component of the responses to saline and alkaline stress in the halophyte Leymus chinensis (Trin.). Plant Growth Regul. 64, 129–139 (2011).

[b35] Fiehn O. . Metabolite profiling for plant functional genomics. Nat. Biotech. 18, 1157–1161 (2000). 10.1038/81137 11062433

[b36] Erb M., Meldan S. & Howe G. A. Role of phytohormones in insect-specific plant reactions. Trends Plant Sci. 17, 250–259 (2012). 22305233 10.1016/j.tplants.2012.01.003 PMC3346861

[b37] Schmelz E. A. . Simultaneous analysis of phytohormones, phytotoxins, and volatile organic compounds in plants. Proc. Natl. Acad. Sci. USA 100, 10552–10557 (2003). 12874387 10.1073/pnas.1633615100 PMC193599

[b38] Lin L. & Tan R. X. Cross-kingdom actions of phytohormones: a functional scaffold exploration. Chem. Rev. 111, 2734–2760 (2011). 21250668 10.1021/cr100061j

[b39] Piotrowska-Niczyporuk A., Bajguz A., Zambrzycka E. & Godlewska-Zylkiewicz B. Phytohormones as regulators of heavy metal biosorption and toxicity in green alga Chlorella vulgaris (Chlorophyceae). Plant Physiol. Biochem. 52, 52–65 (2012). 22305067 10.1016/j.plaphy.2011.11.009

[b40] Penfield S. Temperature perception and signal transduction in plants. New Phytol. 179, 615–628 (2008). 18466219 10.1111/j.1469-8137.2008.02478.x

[b41] Traw M. B. & Bergelson J. Interactive effects of jasmonic acid, salicylic acid, and gibberellin on induction of trichomes in Arabidopsis. J. Am. Soc. Plant Biol. 133, 1367–1375 (2003). 10.1104/pp.103.027086 PMC281631 14551332

[b42] Masoud A. N., Doorenbos N. J. & Quimby M. W. Mississippi-grown *Cannabis sativa* L. IV: Effects of gibberellic acid and indoleacetic acid. J. Pharm. Sci. 62, 316–318 (1973). 4686412 10.1002/jps.2600620230

