# Supplementary material for: Imaging of Endogenous Metabolites of Plant Leaves by Mass Spectrometry Based on Laser Activated Electron Tunneling
Source: Sci Rep. 2016 Apr 7;6:24164. doi: 10.1038/srep24164 (PMC4823709; doi:10.1038/srep24164)
Supplement: Supplementary Information [file srep24164-s1.doc]

**Supporting Information:**

**Imaging of Endogenous Metabolites of Plant Leaves by Mass Spectrometry Based on Laser Activated Electron Tunneling**

**Lulu Huang^+^, Xuemei Tang^+^, Wenyang Zhang, Ruowei Jiang, Disong Chen, Juan Zhang, Hongying Zhong^*^**

**Key Laboratory of Pesticides and Chemical Biology, Ministry of Education, College of Chemistry, Central China Normal University, Wuhan, Hubei 430079, P. R. China**

*** To whom correspondence should be addressed. Email:** [**hyzhong@mail.ccnu.edu.cn**](mailto:hyzhong@mail.ccnu.edu.cn)**, Tel: 86-27-67862616**

**^+^These authors contribute equally to this work**

| **Supplementary Table 1. Putatively identified fatty acids, small organic acids and phytohormones in a *Cayratia japonica* plant leaf with LAET imaging approach** | | | | | |
| --- | --- | --- | --- | --- | --- |
| category | Name and elemental composition | Expt (m/z) | Obsd (m/z) | Error (Da) | Intensity |
| Fatty acids | C15:1(C_15_H_28_O_2_) | 239.2011 | 239.1953 | 0.0058 | 30736 |
|  | C15:0(C_15_H_30_O_2_) | 241.2902 | 241.2948 | 0.0046 | 2924 |
|  | C16:1(C_16_H_30_O_2_) | 253.2168 | 253.2160 | 0.0008 | 844 |
|  | C16:0(C_16_H_32_O_2_) | 255.2324 | 255.2316 | 0.0008 | 28039 |
|  | C17:2(C_17_H_30_O_2_) | 265.2168 | 265.2229 | 0.0061 | 30511 |
|  | C17:1(C_17_H_32_O_2_) | 267.2324 | 267.2365 | 0.0041 | 26480 |
|  | C17:0(C_17_H_34_O_2_) | 269.2481 | 269.2536 | 0.0055 | 12735 |
|  | C18:3(C_18_H_30_O_2_) | 277.2168 | 277.2248 | 0.0080 | 8772 |
|  | C18:2(C_18_H_32_O_2_) | 279.2324 | 279.2411 | 0.0087 | 11352 |
|  | C18:1(C_18_H_34_O_2_) | 281.2481 | 281.2570 | 0.0011 | 5306 |
|  | C18:0(C_18_H_36_O_2_) | 283.2637 | 283.2734 | 0.0097 | 51117 |
|  | C22:6(C_22_H_32_O_2_) | 327.2324 | 327.2255 | 0.0069 | 16558 |
| Small organic acids | Citric acid (C_6_H_8_O_7_) | 191.0192 | 191.0223 | 0.0031 | 56016 |
| Phytohormones | Gibberellic acid(C_19_H_22_O_6_) | 345.1338 | 345.1359 | 0.0021 | 7984 |
|  | Abscisic acid  (C_15_H_20_O_4_) | 263.1283 | 263.1325 | 0.0042 | 19006 |
